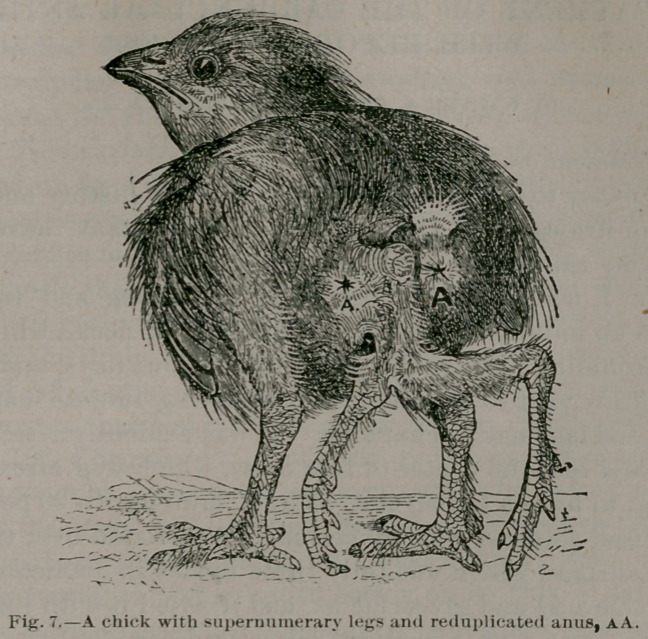# Cephalhæmatoma and Teratomata

**Published:** 1891-05

**Authors:** J. Bland Sutton


### CEPHALHÆMATOMA AND TERATOMATA.

BY J. BLAND SUTTON, F. R. S.

### CEPHALHÆMATOMA.

In November, 1887, Mr. Treves exhibited to the clinical Society a lad with a large swelling on the head. He is described as a "case of cephalhæmatoma with extensive ossification of the cyst-wall." The boy was 11 years of age, and in

Fig. 1.—Boy with an old cephalæmatoma over the right parietal bone (after Silcock.)

perfect health. The swelling was strictly limited to the right parietal bone, and covered with hairy scalp. The central parts were soft, fluctuating, and the seat of feeble pulsation, but the periphery seemed to consist of a crater of hard bone firmly adherent to the skull. At the age of four months this boy fell, his head striking the floor; a bump formed, persisted and increased in size as the boy grew. Upon these facts Treves came to the not unreasonable conclusion that the tumor was primarily a subpericranial cephalhæmatoma, a considerable portion of the wall having subsequently ossified. (Fig. 1.)

In February, 1888, this lad was exhibited to the Society by Mr. Silcock, as an example of traumatic meningocele. A committee reported on the case to the effect that the tumor was probably a cephalhydrocele, "that is, a pulsatile tumor containing cerebro-spinal fluid communicating with the interior of the skull by an abnormal opening.

Thus vol. xxi. of the *Transactions of the Clinical Society* contains a case twice exhibited by competent surgeons, each holding a widely different opinion as to its nature. It has long been known that a cephalmhæmatoma (a collection of blood, extravasated in consequence of injury, between the vault of the skull and pericranium) may ossify around the edges and give rise to the motion of a depressed fracture.

Fig. 2.—Monkey (Cebus monachus) with a large cephalhæmatoma.

In 1889 a monkey (*Cebus monachus*) was deposited in the Zoological Gardens. On the top of its skull was a large rounded tumor, nearly as large as its head. The tumor was soft and fluctuating at the top, and a feeble pulsation could be felt. The part near the skull was extremely hard and felt like bone. The monkey was in excellent health and spirits, in no way encumbered by its burden. It continued in this way for many weeks, and the tumor did not increase in size, but the hardening of the walls became more extensive. Some months later the monkey was evidently in a bad way, emaciated, in pain, and suffering from thirst and fever. The tumor had not increased in size, but the hardening now involved the greater part of the walls of the tumor, the summit still remaining soft. As the animal seemed to be in great suffering, it was killed by means of chloroform. The tumor was filled with fluid blood; the walls were formed by the pericranium, the greater portion of which had ossified; it was, in fact, an old cephalhæmatoma. The pressure of the fluid had induced thinning of that portion of the skull which formed the floor of the hæmatoma.

The specimen is interesting as showing the length of time blood may remain fluid when extravasated. The monkey lived in the Zoological Gardens seven months, and the tumor was present when it entered the menagerie. It is also valuable—at least it seems so to me—for the light it sheds on Treve's case, and indicates that the tumor in the boy was, as Treves believed, a cephalhæmatoma with the walls extensively ossified.

TERATOMATA.

Louise L., known as the *dame a quartre jambes*, has a pair of supernumerary hind limbs attached to the pubic symphisis, as represented in Fig. 3. She married at the age of 14 years and 9 months, and is the mother of two well formed children. The autosite cannot initiate any movement in the accessory legs, although she can readily localize the prick of a pin made on any part of the parasite; she is also uncomfortable when the parasite is cold. A fact of some interest is the existence of a furrow between the buttocks belonging to the supernumerary legs. In this furrow is an imperforate fossa representing the anus of the parasite; it is situated about 12 centimetres from the vulva of the autosite. I draw particular attention to this, as it is of some importance.

The next variety is illustrated by Jean Baptiste dos Sontos, of Portugal. The child has got a median unpaired leg projecting from the pubes and situated between the normal legs. The distal extremity is furnished with nine separate digits, but the median one consists of two coalesced big toes. On the part of this leg corresponding to the buttock is a dimple representing the imperforate anus of the parasite, and what is more interes.ing, the child has two separate and, as determ; ined in later life, functional penes. We have no evidence that he

Fig. 3. -Louise L , dame a quartre jambes (after Ed. Bugnion.)

has more than one pair of testes, a point which it is hoped will some day be clearly established, and it will be valuable evidence in connection with the origin of these malformations.

The third variety is illustrated in the girl sketched in Fig. 5. Over the lower end and posterior aspect of the sacrum is an irregular lobulated tumor, with a depression indicating an imperforate ano-genital orifice. Projecting from the left side of this mass is an ill-shaped leg, the foot of which is in the posi-

Fig. 4.—Jean Baptiste dos Santos, at the age of 6 months.

tion of talipes equino-varus.

The remarkable condition represented by the three speci-

Fig. 5.—A sacral teratoma with supernumerary leg.

mens just described are examples of dichotomy affecting the posterior extremity of the axis of the trunk. When dichotomy affects the cephalic portion of the trunk we get reduplication, wholly or in part, of the head, and, when extensive, reduplication of the anterior pair of limbs. It naturally follows in posterior cleavage that the pelvic girdle and associated limbs are reduplicated, as well as the nether extremity of the gut, and I have been careful to point out that in three at least of my type cases a dimple, representing an imperforate anus, was detected.

So long as sacral teratomata were studied in man alone they were regarded as mysterious or curious. Comparative path-

Fig. 6.—A chick with supernumerary legs.

ology throws much valuable light on this obsurity. When we find a series of manifestations running through all the more important groups of animals from lobsters, worms, star-fish, fish, amphibian reptiles, birds, and mammals up to man, we may feel certain that some common cause underlies their production.

Fig. 7.—A chick with supernumerary legs and reduplicated anus, AA.

Looking broadly at the group of congenital sacro-coccygeal tumors, it would be idle to deny that a knowledge of their pathology is of the utmost service, even to those surgeons who boast that they are "eminently practical," and affect to despise the scientific side of our art.—*British Medical Journal*, Feb. 14. —*New York Medical Abstract.*

## Figures and Tables

**Fig. 1. f1:**
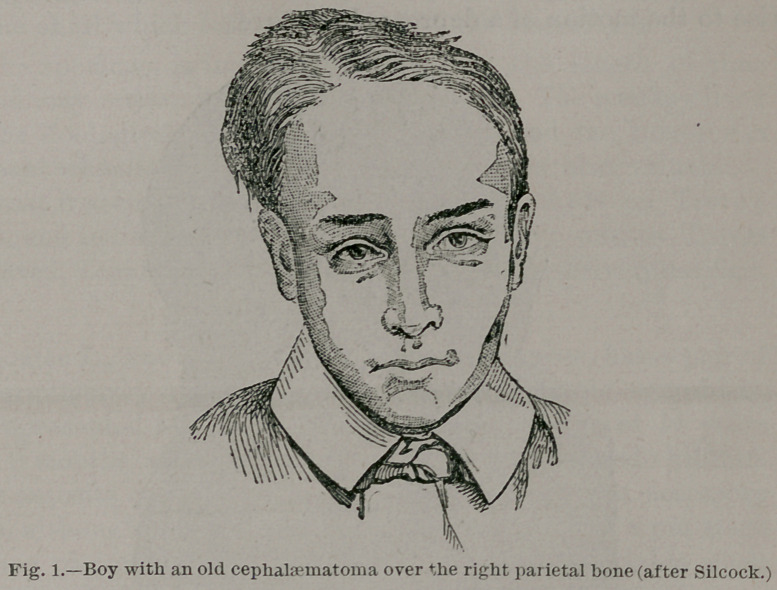


**Fig. 2. f2:**
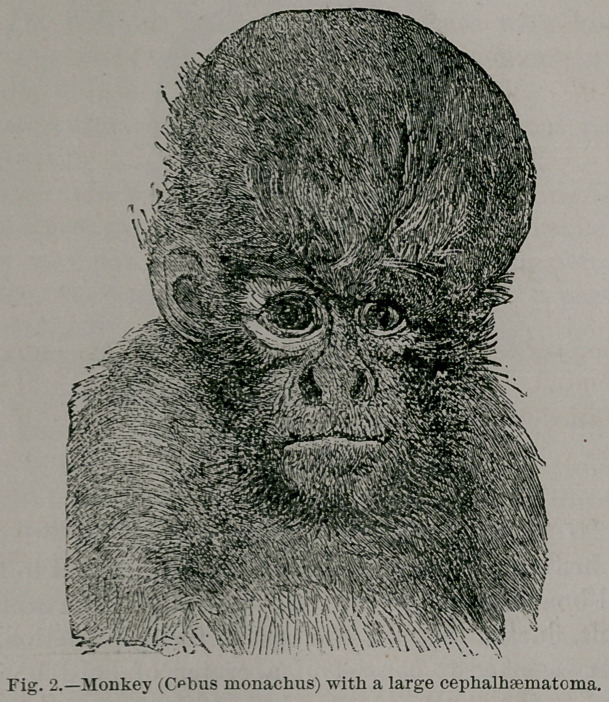


**Fig. 3. f3:**
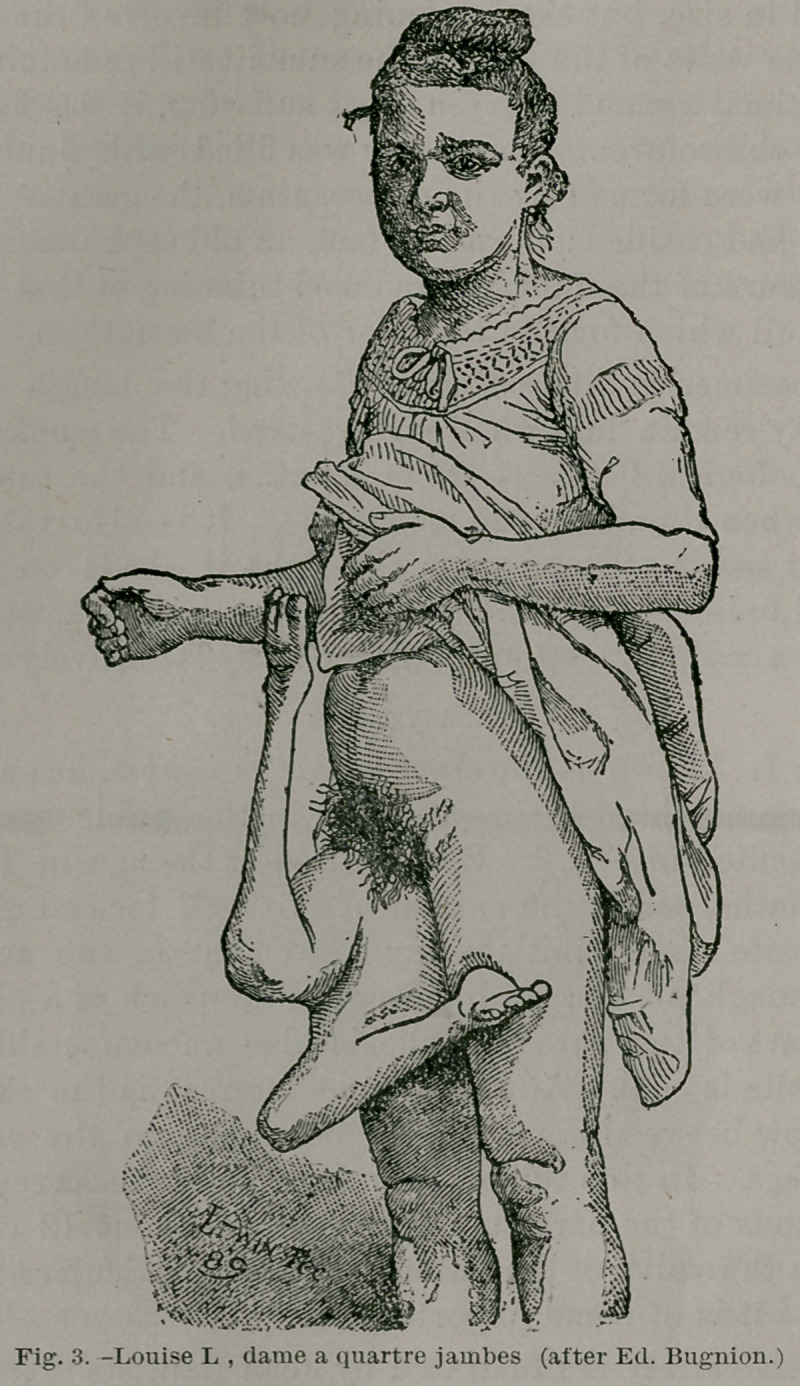


**Fig. 4. f4:**
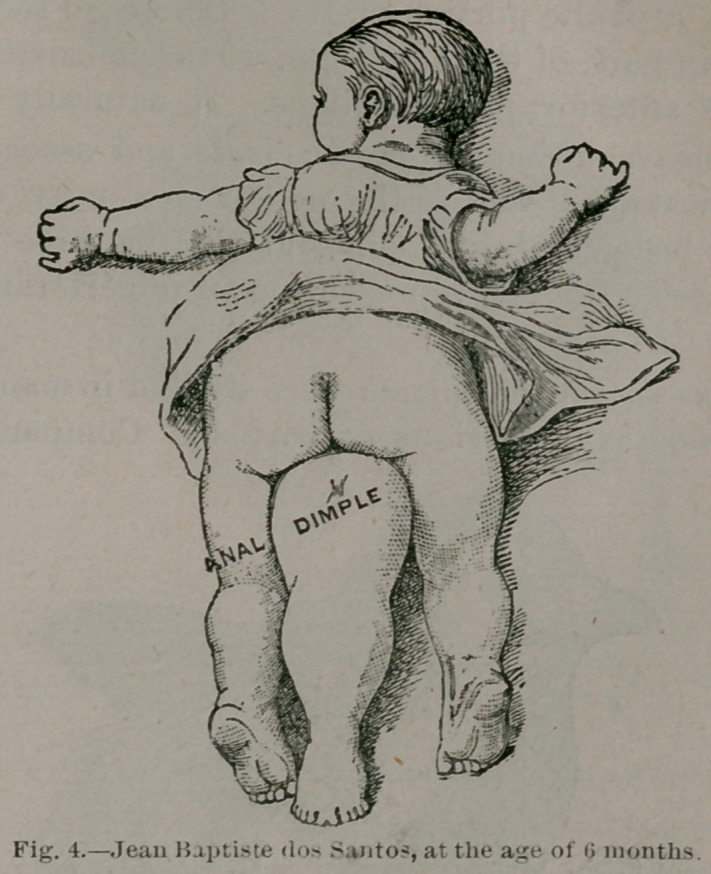


**Fig. 5. f5:**
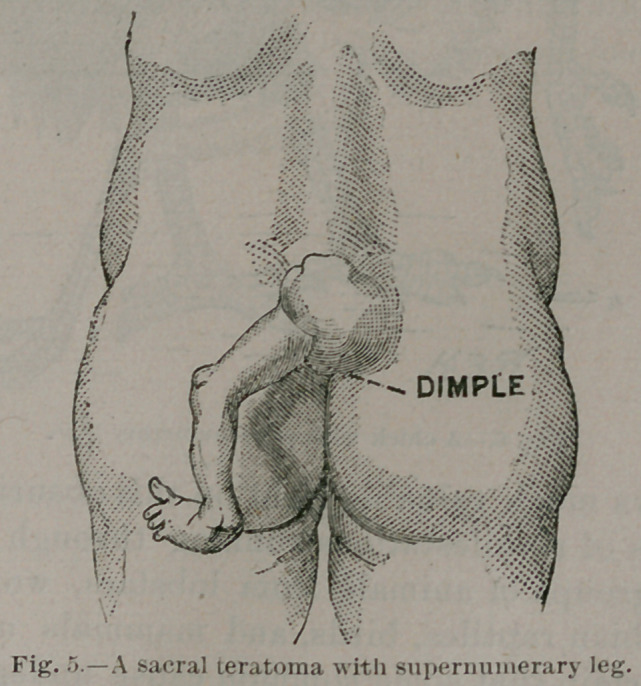


**Fig. 6. f6:**
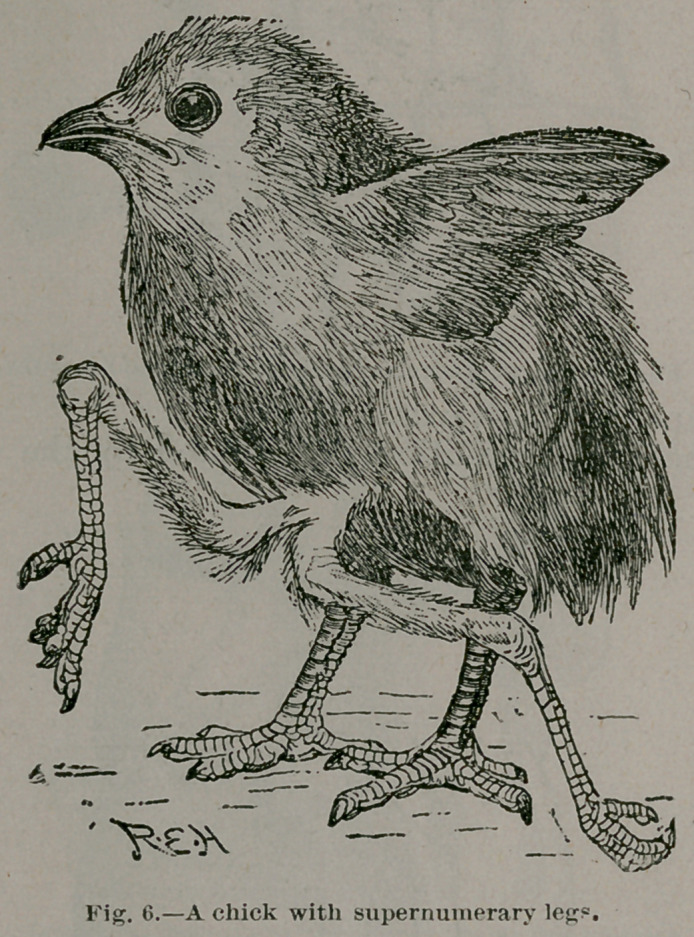


**Fig. 7. f7:**